# BRAF^V600E^ status and clinical characteristics in solitary and multiple papillary thyroid carcinoma: experience of 512 cases at a clinical center in China

**DOI:** 10.1186/1477-7819-10-104

**Published:** 2012-06-08

**Authors:** Xiangqian Zheng, Tingting Xia, Lin Lin, Songyuan Gao, Yigong Lee, Yang Yu, Songfeng Wei, Ming Gao

**Affiliations:** 1 Oncology Key Laboratory of Cancer Prevention and Therapy, Tianjin Medical University Cancer Institute and Hospital, Tianjin, P. R. China; 2 Department of Pathology, Tsinghua University Medical College, Tsinghua University, Beijing, P.R.China; 3 Department of Thyroid and Neck Tumor, Tianjin Medical University Cancer Hospital and Institute, Huanhuxi Road, Ti-Yuan-Bei, Hexi District, Tianjin, 300060, P.R. China

**Keywords:** Papillary thyroid carcinoma, Multiple PTC, Solitary PTC, BRAF^V600E^ mutation

## Abstract

**Background:**

Papillary thyroid carcinoma (PTC) is one of the most frequent endocrine malignancies. In most cases, it often presents as multifocal tumor. It has been reported that multifocal tumors are associated with elevated risk of lymph node and distant metastases. Multifocality is also one of the factors predicting prognosis. Recent studies show that BRAF^V600E^ mutation occurs more frequently in aggressive PTC. The purpose of this study was to evaluate BRAF^V600E^ status and clinicopathological features in multiple and solitary PTC.

**Methods:**

We performed a retrospective study to analyze 512 PTC cases who received surgery, including 376 solitary PTCs and 136 multiple PTCs.

**Results:**

Multiple PTC is more related to lymph node metastasis and vascular invasion than solitary PTC. However, the distant metastasis rate and 10-year survival rate showed no difference between these two groups. BRAF^V600E^ mutation status was more frequent in multiple PTC patients with lymph node metastasis and late stage at diagnosis.

**Conclusion:**

BRAF^V600E^ mutation is most commonly associated with extra-thyroidal extension and lymph node metastasis in PTC. Multiple PTC patients with young age, large tumors and BRAF^V600E^ mutation should be followed carefully. Our study provides useful information for PTC patients’ followup and treatment.

## Background

Thyroid cancer is the most common endocrine neoplasm, and one of the leading causes of death in patients with endocrine cancers [1,2]. The incidence of thyroid cancer is increasing more rapidly than other cancers in both the United States [3] and other countries [4]. Papillary thyroid carcinoma (PTC) is the most frequent type of thyroid cancer and represents one of the most frequent endocrine malignancies [5-7], it originates from the follicular cells of the thyroid. Surgery can cure most well-differentiated thyroid cancer when diagnosed and treated in early stage before the establishment of local or distant metastases. However, there are still some patients with well-differentiated thyroid cancer that die of the disease.

PTC is often present as multiple tumors [8,9]. Postsurgical pathologic analysis has shown that 18% to 87% of PTC have multiple noncontiguous tumor foci in the individual glands, with a dominant tumor and multiple additional smaller foci of microcarcinomas [10,11]. Studies have also reported that “multiple” tumors arising from metastasis of a single primary carcinoma origin, and “multicentricity” tumors arising independently from different origins in a context of genetic and/or environmental predisposition [12-14]. Some clinical factors have been used to predict which patients with thyroid cancer might have worse prognosis. Factors such as older age (>45 years old), male gender, certain histologic subtypes (i.e. tall cell, columnar cell and diffuse sclerosing variants of PTC), tumor size greater than 4 cm and presence of extrathyroidal extension are associated with neck recurrences and distant metastases [15-18]. In addition, multiple PTC is associated with increased risks of metastases and regional recurrences [19,20]. Intra-glandular dissemination from the dominant tumor may serve as an indicator of metastatic potential and more aggressive phenotype.

The B-type Raf kinase (BRAF) mutation is the most common genetic alteration in papillary thyroid cancer (PTC). Of the three forms of Raf kinase, BRAF is the most potent activator of the mitogen-activated protein kinase (MAPK) pathway, which plays a major role in the regulation of cell growth, division, and proliferation [21]. An activating mutation of BRAF^V600E^ has been found in 36% to 69% of patients with PTC. This mutation is very prevalent in PTC and is frequent in the tumors in late stages (stages III and IV) of PTC [22,23], with lymphovascular invasion and metastases [24,25]. However, other studies did not find any association between BRAF^V600E^ mutation and tumor stage, local invasiveness and lymph node metastasis [26].

The purpose of this study was to evaluate the clinicopathologic features with multiple and solitary PTC, and found that the status of BRAF^V600E^ mutation in multiple PTC and solitary PTC.

## Methods

### Patients

512 patients with PTC underwent surgery from May 1995 to Jan 2000 at Tianjin Medical University Cancer Institute and Hospital. The diagnosis of PCT was pathologically confirmed in all patients (Table 1). 376 patients (281 female and 95 malewith mean age of 42.5 ± 17.4, 4–78 years old) had solitary PTC. 136 patients (104 female and 32 male, with mean age of 44.3 ± 15.1, 6–76 years old) had multiple PTC, including 79 patients with 2 separated tumor foci, 28 patients with 3 tumor foci, 29 patients with four or more tumor foci. Thus, we investigated a total of 369 PTC foci in 136 multiple PTC patients.

**Table 1:** Clinicopathological Characteristics of 512 cases of PTCs

**Characteristics**	**Number**
Number of patients	512
Male/female	127/385
Mean age (yr) ± SD	43.5 ± 16.4 (4–78)
Mean size (cm) ± SD	1.58 ± 1.35
Less than 1 cm	235 (45.9%)
1 cm - 2 cm	173 (33.8%)
2 cm – 4 cm	73 (14.3%)
More than 4 cm	31 (6.0%)
Multiple	136 (26.6%)
Extra-thyroidal invasion	128 (25.0%)
Lymph node metastasis	207 (40.4%)
Distant metastasis	20 (3.9%)
Recurrence	36 (7.0%)
Family history	13 (2.5%)
BRAF^v600E^ mutation	263 (51.4%)
TNM stage	
I	242 (47.3%)
II	151 (29.5%)
III	83 (16.2%)
IV	36 (7.0%)

The TNM stage is based on the AJCC Cancer Staging Manual, 7^th^ edition (2002).

### Treatment and follow-up of PTC

The patients were treated with thyroidectomy in the Department of head and neck. The initial treatment was lobectomy and total/ near total thyroidectomy, central neck and/or lateral-cervical lymph node dissection when necessary,. (According to the National Comprehensive Cancer Network (NCCN), 1995) Tumor samples were obtained in accordance with protocols approved by the institutional review board, and informed consent was achieved 1 day before surgery together with the surgical one.

Histological diagnosis was made independently, in a blinded fashion, by two pathologists. Tumors were classified according to the histopathological typing of the World Health Organization. The multifocal or solitary tumor was identified by pathologist. A concordance rate of 98% was obtained between the two pathologists. The few discordant cases were discussed with a third pathologist.

^131^I postoperative administration was performed for thyroid remnant ablation when the patient received total or near-total thyroidectomy, L-thyroxine (L-T4) suppressive therapy. Additional ^131^I therapies were given to treat local recurrences or distant metastases that were not removable by surgery. The patients was performed cervical ultrasound and TSH-suppressed thyroglobulin 6 months after treatment, the anti-throglubulin antibody and T3, T4, TSH, serum thyroglobulin was measured after thyroxine withdrawal or rhTSH stimulation approximately 12 months after the ablation to verify absence of disease.

Criteria for disease remission were negative for ^131^I Whole Body Scanning (WBS) and Tg < 2 ng/mL after L-T4 withdrawal or recombinant thyroid-stimulating hormone injection. The diagnosis of recurrence was made after observing an elevation of Tg levels associated with focal areas of ^131^I uptake at WBS and/or evidence of lesions at ultrasound or computed tomography (CT) and/or positive cytology examination. 435 patients were followed up by reviewing the clinical records for 10 years or to the death of patients. 46 patients were dead in 10 years, 77 patients were lost of follow up.

### Tumor sample preparation and examination

Specimens from surgery were cut and fixed in 10% formalin for preparing paraffin-embedded sections and stained with hematoxylin and eosin (H & E) for histologic examination. One to 3 representative sections of the tumor and all suspicious lesions were submitted for microscopic examination by two endocrine pathologists. The diagnosis of PTC was based on characteristic architectural features, including the presence of true papillae and/or characteristic nuclear changes, such as ground glass nuclei, nuclear pseudoinclusions, and nuclear grooves.

### Detection of BRAF^V600E^ mutation

DNA was extracted from paraffin-embedded tissue. Briefly, unstained tumor tissues on 20-mm-thick sections were chosen by comparing to H & E-stained sections. For larger tumors, the marked areas were deparaffined and tissues were collected. For small tumors, laser-capture microdissection was performed to collect tissues. Samples from large tumors were incubated in TE9 (0.5 M TRIS, 0.2 M EDTA, 0.01 M sodium chloride, and 1% sodium dodecyl sulfate; pH 9.0) and 0.2 mg/ml of proteinase K for 4 days at 55°C. Small tumor samples in Laser-capture microdissection caps were incubated in TE9 for 2 days at 37°C. Fresh proteinase K was added daily. Samples were centrifuged and supernatants were subjected to digestion for two additional days at 55°C. Chelex 100 resin (Bio-Rad) was added to each sample and incubated for 1 hour and the supernatant was removed. DNA was extracted using phenol–chloroform and concentrated by ethanol precipitation. DNA was resuspended in TRIS–EDTA (10 mM TRIS hydrochloride and 1 mM EDTA; pH 8.0).

DNA samples were applied for PCR analysis using the following primers: BRAF 11F (5′-TCCCTCTCAGGCATAAGGTAA-3′) and BRAF 11R (5′-CGAACAGTGAATATTTCCTTTGAT-3′; PCRproduct, 312 bp) for exon BRAF11, and primers BRAF 15F (5′-TCATAATGCTTGCTCTGATAGGA-3′) and BRAF 15R (5′-GGCCAAAAATTTAATCAGTGGA-3′; PCR product, 223 bp) for exon BRAF 15.

Cycle sequencing of the purified PCR products was performed by using one of the PCR primers and the big dye terminator sequencing kit (Applied Biosystems, Foster City, CA). The Sephadex G-50–purified cycle sequencing products were analyzed using an ABI PRISM 310 Genetic Analyzer (Applied Biosystems).

### Statistical analysis

Statistical analyses were performed with SPSS software (version 11.0; SPSS Inc., Chicago, IL). Chi-square or Fisher exact tests were used to compare frequencies between groups. All data were expressed as means ± SD. Differences between group means were compared by the independent sample Student t-test or the Mann–Whitney U-test. P value < 0.05 was considered statistically significant.

## Results and discussion

### Comparison of progression between patients with solitary and multiple PTC

Papillary thyroid carcinomas represent about 90% of all thyroid cancers and tumor incidence has been increasing in recent decades [2,10]. Although PTC patients generally respond in a favorable manner and have a favorable prognosis, many develop recurrence and some die from this disease. Pathological analysis of patients with PTC undergoing surgical treatment have shown that the incidence of multiple noncontiguous tumor foci in individual glands is high [27,28]. Thus, we evaluated progression of disease between patients with solitary and multiple PTC.

Large tumor size, age, gender, extra-thyroidal invasion, lymph node and distant metastasis, and the variant of the PTC are the main determinants for poor prognosis in PTC patients.

**Figure 1:**
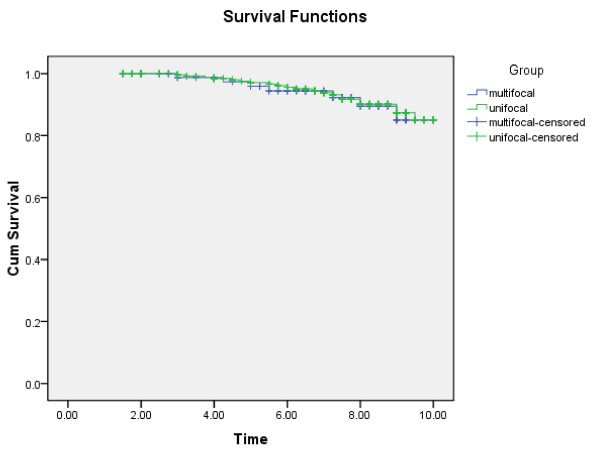
Comparison of 10-year survival rate between solitary and multiple PTC.

We performed a retrospective study of 376 patients with solitary PTC and 136 patients with multiple PTC to assess the risk factors in disease progression at diagnosis, including sex, age at diagnosis, extra-thyroidal extension, vascular invasion, lymph node metastasis, distant metastasis (including lung, bone and liver) Hashimoto’s thyroiditis, and 10-year survival rate, etc (Table 2). Our results showed that the Micro variant, rate of lymph node metastasis, and tumor recurrence in patients with were significantly higher in patients of multiple PTC than those with solitary PTC (p < 0.001). Tumor size, vascular invasion, incidence of Hashimoto’s thyroiditis, and having family history were also higher in patients with multiple PTC compared to those in patients with solitary PTC (p < 0.05). However, other clinical parameters that we evaluated showed no significant differences between these two groups. The 10-year survival rate between multiple PTC and solitary PTC has no significant difference (Figure 1).

**Table 2:** Comparison of progression between patients with solitary and multiple PTC

	**Multiple PTC**	**solitary PTC**	**P value**
Number	136	376	
Male/female	32/104	95/281	0.451
Mean age (yr) ± SD	44.3 ± 15.1	42.5 ± 17.4	0.226
Mean size (cm) ± SD	1.29 ± 1.44	1.67 ± 1.15	0.048
Micro variant	84 (61.8%)	147(39.1%)	< 0.001
Tall cell variant	17 (12.5%)	38 (10.1%)	0.267
Extrathyroidal invasion	35 (25.9%)	93 (24.7%)	0.450
Vascular invasion	15 (11.0%)	16 (4.3%)	0.006
Hashimoto’s thyroiditis	36 (26.5%)	61 (16.2%)	0.007
Lymph node metastasis	74 (54.4%)	123 (32.7%)	< 0.001
Distant metastasis	6 (4.4%)	14 (3.7%)	0.447
Recurrence	21 (15.4%)	15 (4.0%)	< 0.001
10-year survival rate	125 (91.9%)	341 (90.7%)	0.579
BRAF^v600E^ mutation	75 (55.1%)	188 (50%)	0.176
Family history	7 (5.1%)	6 (1.6%)	0.032
TNM stage			
I	56 (41.2%)	186 (49.5%)	
II	40 (29.4%)	111 (29.5%)	0.031
III	24 (17.6%)	59 (15.7%)	
IV	16 (11.8%)	20 (5.3%)	

The TNM stage is based on the AJCC Cancer Staging Manual, 7^th^ edition (2002).

Lymph node metastasis, as a sign of high aggressiveness, has been reported to be a risk factor for persistence/recurrence of disease and also for cancer-related mortality in many studies [29-31]. Our data show that multiple PTC correlates with higher lymph node metastasis. We analyzed clinical parameters in multiple PTC patients in the presence or absence of lymph node metastasis.(Table 3) We found that the younger age, larger tumor size, extra-thyroidal extension, vascular invasion and distant metastases have an significant association with lymph node metastases in multiple PTC (p < 0.05).

**Table 3:** Clinical parameters in multiple PTC patients in the presence or absence of lymph node metastasis

**Lymph node metastasis**	**Yes**	**No**	**P value**
Number	74 (54.4%)	62 (45.6%)	
Age (years)	41.5 ± 12.2 (9–60)	50.3 ± 14.2 (18–75)	0.028
Tumor diameter (mm)	2.08 ± 1.51	1.13 ± 1.36	0.016
Extrathyroidal invasion	26 (35.1%)	9 (14.5%)	0.005
Vascular invasion	13 (17.6%)	2 (3.2%)	0.007
Hashimoto’s thyroiditis	23 (31.1%)	13 (21.0%)	0.128
Distant metastasis	6 (8.1%)	0 (0%)	0.024

In patients with surgical treatment for PTC, pathological analysis commonly identifies multiple noncontiguous tumor foci in individual glands. Estimates of the frequency of such multiple tumors vary, between 18 and 87 percent depending on the techniques used. Multiple tumors have been associated with increased risk of lymph node and distant metastases, persistent local disease after initial treatment, and regional recurrence [32]. Therefore, our results are consistent with previous reported studies that patients with multiple PTC associate with increased risk of lymph-node metastases and regional recurrence. However, in this study, distant metastasis did not significantly differ. All these findings suggest that patients with multiple papillary thyroid cancer should receive aggressive treatment. According to the guideline by American Joint Committee on Cancer (AJCC) (Table 2). An association can be made when comparing tumor stage in patients with solitary and multiple PTC. More stage III/IV cases were found in multiple PTC patients compared to those in solitary PTC patients. Consistently, the number of patients with stage I is higher in solitary PTC patients than that in multiple PTC.

### BRAF^V600E^ mutational status and clinicopathologic characteristics in 512 patients of PTC

BRAF^V600E^ is the most prevalent genetic alteration implicated in the initiation and progression of PTC, especially in aggressive subtypes such as the tall cell variant of PTC and in those with extra-thyroidal extension and lymph node or distant metastases. The mutation has been associated with both radioactive iodine refractoriness and PTC recurrence. Xing et al. [33,34] have reviewed studies of the association between the BRAF^V600E^ mutation and clinicopathologic characteristics in PTC. In a review of 12 studies with a total of 1,168 patients. Lee et al. [35,36] reported that the BRAF^V600E^ mutation was detected in 49% (570/1,168) cases. Recent studies report a varying prevalence of the mutation in PTC, ranging from 29 to 83%.

Correlation analysis between the BRAF^V600E^ mutation status and the clinicopathologic characteristics of PTC patients revealed that extra-thyroidal invasion, central and lateral lymph node metastasis were significantly higher when BRAF^V600E^ mutation is present(p < 0.001). Elder age and male gender are much more prevalent in BRAF^V600E^ mutation patients (p < 0.05). However, the BRAF^V600E^ mutation was not significantly associated with multiple PTC and distant metastasis (Table 4).

**Table 4:** **BRAF**^**V600E**^**mutational status in 512 patients of PTC**

**Characteristic**	**BRAF^V600E^ mutation**		**P value**
	**Positive**	**Negative**	
Number	263	249	
Age (yr)	45.4 ± 12.3	40.8 ± 16.2	0.028
Male/ Female ratio	79/184	48/201	0.003
Extrathyroidal invasion	91 (34.6%)	37 (14.9%)	< 0.001
Multiple PTC	75 (28.5%)	61 (24.5%)	0.184
Central LNM*	151 (57.4%)	56 (22.5%)	< 0.001
Lateral LNM*	83 (31.6%)	31 (12.5%)	< 0.001
Distant metastasis	18 (6.8%)	12 (4.8%)	0.261

LNM: lymph node metastases.

Many other studies have found that the BRAF^V600E^mutation is most commonly associated with extra-thyroidal extension, lymph node metastasis, and advanced disease stage. [37] Our data also found a significant association between the BRAF^V600E^ mutation and extra-thyroidal invasion and lymph node metastasis. This study also showed that the BRAF^V600E^ mutation correlated with male gender and elder age.

We also compared clinicopathological characteristics and BRAF^V600E^ mutation status between solitary and multiple PTC. (Table 5) No significant difference in age, extra-thyroidal extension, lymph node metastasis and distant metastasis were found between solitary and multiple PTC with BRAF^V600E^ mutations, beside the male gender.

**Table 5:** **Correlation between clinicopathological characteristic and BRAF**^**V600E**^**mutation in solitary PTC and multiple PTC**

**Characteristic**	**BRAF^V600E^ mutation patients**		
	**solitary PTC (188)**	**multiple PTC (75)**	**P value**
Age (yr)			
Less than 45	67 (35.6%)	30 (40%)	0.3
45 or older	121 (64.4%)	45 (60%)	
Gender			
Male	62 (33.0%)	17 (22.7%)	0.045
Female	126 (67.0%)	58 (77.3%)	
Extrathyroidal invasion			
Yes	66 (35.1%)	25 (33.3%)	0.451
No	122 (64.9%)	50 (66.7%)	
Lymph node metastasis			
Yes	104 (55.3%)	47 (62.7%)	0.171
No	84 (44.7%)	28 (37.3%)	
Distant metastasis			
Yes	13 (6.9%)	5 (6.7%)	0.591
No	175 (93.1%)	70 (93.3%)	

### Comparison of the effects of BRAF^V600E^ mutation on progression in multiple PTC patients

The multiple foci in papillary thyroid carcinoma is a common clinical finding, but the origin of these foci is ambiguous. Despite attempts to establish whether multiple intra-thyroidal tumors are metastases of a primary thyroid tumor or arise independently, the question remains unresolved. Evidence from previous studies has lent support to both arguments. We analyzed whether multiple intra-thyroidal tumors were metastases of a primary thyroid tumor cell or arise independently from different tumor cells, and effects of tumor foci origin on disease progression. We assessed the origin of multiple PTC by analyzing the BRAF^V600E^ mutation status in the multiple PTC. The status of the BRAF^V600E^ mutation was heterogeneous (35 mixed BRAF^V600E^ mutation status of 75 BRAF^V600E^ mutation multiple PTC) in 46.7% cases, suggesting at least some of multiple PTC arise as independent tumors. It’s difficult to study whether those with same BRAF change are from the same origin, since BRAF is a hot spot mutation and many cases have the same change just by chance. It should be noted that the possibility cannot be excluded that tumors with or without BRAF^V600E^ mutation in PTC have a unique clonal origin, because BRAF^V600E^ mutation is a common genetic alteration in PTC and more than one tumor foci may have this mutation at the same time. In our study, 46.7% multiple PTC are heterogeneous.

Studies have showed that BRAF^V600E^ mutation was significantly more frequent in multiple PTC patients with extra-thyroid invasion, and lymph nodes metastasis. These observations indicate that BRAF^V600E^ mutation may be a predictor of tumors with high aggressiveness [38-40]. Therefore, we evaluated the status of BRAF^V600E^ mutation in progression of multiple PTC patients. In these 136 patients with multiple PTC, 40 patients (29.4%) had BRAF^V600E^ mutation in all foci, 61 patients (44.9%) patients showed negative for BRAF^V600E^ mutation in all foci, and 35 patients (25.7%) showed mixed status for BRAF^V600E^ mutation (BRAF^V600E^ mutation positive and negative tumor foci coexisted in the same patient). When we analyzed BRAF^V600E^ mutation status in multiple PTC, we found that BRAF^V600E^ mutation mixed status was more frequent in patients with more than 4 tumors compared with patients with 2 or 3 tumors.

Furthermore, our results demonstrated that the BRAF^V600E^ mutation was significantly associated with lymph node metastasis and late age at diagnosis (≤14 year old in patients) with multiple PTC. However, there was no difference in tall cell variant, extra-thyroidal invasion, vascular invasion, Hashimoto’s thyroiditis, distant metastasis and 10-year survival rate between multiple PTC patients with or without BRAF^V600E^ mutation (Table 6).

**Table 6:** **The effects of BRAF**^**V600E**^**mutation on progression in multiple PTC patients**

**BRAF^v600E^ mutation**	**All negative**	**All positive**	**Mixed**	**P value**
Number	61	40	35	
Age ≤ 14	15 (24.6%)	1 (2.5%)	1 (2.9%)	0.001
Tall cell variant	3 (4.9%)	8 (20.0%)	6 (17.1%)	0.597
Extrathyroidal invasion	10 (16.4%)	17 (42.5%)	8 (22.9%)	0.214
Vascular invasion	3 (4.9%)	8 (20.0%)	4 (11.4%)	0.097
Hashimoto’s thyroiditis	12 (19.7%)	14 (35.0%)	10 (28.6%)	0.088
Lymph node metastasis	27 (44.3%)	32 (80.0%)	15 (42.9%)	< 0.001
Distant metastasis	1 (1.6%)	3 (7.5%)	2 (5.7%)	0.065
10-year survival rate	56 (91.8%)	35 (87.5%)	34 (97.1%)	0.336

## Conclusion

In conclusion, we showed in this study that late stage tumors, lymph node metastasis and vascular invasion in multiple PTC patients are more frequent than those in solitary PTC patients. In patients with multiple PTC, the status of lymph node metastasis was related to the early age at diagnosis, the larger tumor, extra-thyroidal invasion, and BRAF^V600E^ mutation status. Therefore, multiple PTC patients with young age, large tumors and BRAF^V600E^ mutation should be treated and follow-up carefully.

## Abbreviations

PTC: Papillary thyroid carcinoma.

## Competing interest

The authors declare that there is no conflict of interest for this study.

## Authors’ contributions

Study concepts: XZ Study design: MG. Data acquisition: TX. **A**lgorithms: SW. Data analysis and interpretation: XZ, SG. Statistical analysis: YL, LL. Manuscript preparation: XZ, YY. Manuscript editing: XZ, MG. Manuscript review: MG. All authors read and approved the final manuscript.
